# Automatic Infants’ Pain Assessment by Dynamic Facial Representation: Effects of Profile View, Gestational Age, Gender, and Race

**DOI:** 10.3390/jcm7070173

**Published:** 2018-07-11

**Authors:** Ruicong Zhi, Ghada Zamzmi Dmitry Goldgof, Terri Ashmeade, Yu Sun

**Affiliations:** 1 School of Computer and Communication Engineering, University of Science and Technology Beijing, Beijing 100083, China; 2 Beijing Key Laboratory of Knowledge Engineering for Materials Science, Beijing 100083, China; 3 Department of Computer Science and Engineering, University of South Florida, Tampa, FL 33620, USA; ghadh@mail.usf.edu (G.Z.); goldgof@mail.usf.edu (D.G.); yusun@mail.usf.edu (Y.S.); 4 College of Medicine Pediatrics, University of South Florida, Tampa, FL 33620, USA; tashmead@health.usf.edu

**Keywords:** infants’ pain assessment, dynamic facial representation, profile view, gestational age, gender, race

## Abstract

Infants’ early exposure to painful procedures can have negative short and long-term effects on cognitive, neurological, and brain development. However, infants cannot express their subjective pain experience, as they do not communicate in any language. Facial expression is the most specific pain indicator, which has been effectively employed for automatic pain recognition. In this paper, dynamic pain facial expression representation and fusion scheme for automatic pain assessment in infants is proposed by combining temporal appearance facial features and temporal geometric facial features. We investigate the effects of various factors that influence pain reactivity in infants, such as individual variables of gestational age, gender, and race. Different automatic infant pain assessment models are constructed, depending on influence factors as well as facial profile view, which affect the model ability of pain recognition. It can be concluded that the profile-based infant pain assessment is feasible, as its performance is almost as good as that of the whole face. Moreover, gestational age is the most influencing factor for pain assessment, and it is necessary to construct specific models depending on it. This is mainly because of a lack of behavioral communication ability in infants with low gestational age, due to limited neurological development. To our best knowledge, this is the first study investigating infants’ pain recognition, highlighting profile facial views and various individual variables.

## 1. Introduction

Healthcare for infants in a Neonatal Intensive Care Unit (NICU) is critical for survival; however, the hospitalization stage affects their neurodevelopment and future growth. Invasive medical interventions are often required in clinical treatment, and as a result, infants suffer from repeated procedural pain as part of their general care in the NICU. The early exposure to painful procedures can have negative short and long-term effects on cognitive, neurological, and brain development [1]. Therefore, it is important to judge and measure pain procedures for infants, especially preterm infants under special medical care.

Accurate pain assessment is comprehensive and multidimensional, and the commonly used pain measurements include contextual, behavioral, and physiological tools [2]. Pain is subjective experience, which may easily be affected by physical, cognitive, emotional state, and pain history [3,4]. Infants exhibit pain-related facial activities [5], body movements [6], physiological responses [7], and cortical activities [8] during painful procedures. A self-reported pain assessment method is not applicable for infants with communicative impairment. Multidimensional indicator-based scales are commonly used for infants’ pain assessment, such as Neonatal Infant Pain Scale (NIPS) [9], Face, Legs, Activity, Crying, and Consolability (FLACC) [10], Neonatal Facial Coding System (NFCS) [11] for acute pain assessment, Neonatal Pain, Agitation, and Sedation Scale (N-PASS) [12], Neonatal Pain and Discomfort Scale (EDIN) [13], and Crying, Requires *O*_2_, Increased VS, Expression, and Sleepless (CRIES) [14] for chronic pain assessment.

The observational measures are based on several different behavioral indicators (e.g., facial expression, crying, body activity, and sleeping state) and physiological indicators (e.g., breathing pattern, heart rate, and oxygen saturation) related to pain. Since infants cannot communicate in words, observational measures are commonly preferred and considered as the gold-standard for pain assessment [15]. Although the indicator-based scales are easy to use in practice, the evaluation requires professional caregivers with plenty of training, and manual pain assessment is time-consuming and laboring for long-term continuous pain monitoring, as the caregiver has to assess infants’ pain at short intervals. Moreover, such measures are easily disrupted by the observer’s bias, and various influence factors such as clinical experience, underestimation of pain [16], background, and culture [17,18]. With the rapid development of machine learning and artificial intelligence, an automatic infant pain assessment system is desired for objective and accurate pain assessment.

## 2. Related Work and Contributions

There has been an increasing interest in understanding individual behavioral responses to pain based on facial expressions [19,20,21,22,23], body or head movements [24,25], and sound signals (crying) [26,27,28]. Pain-related behavior analysis is non-invasive and easily acquired by video recording technique. Evidence supports the fact that facial expression is the most specific indicator and is more salient and consistent than other behavioral indicators [29,30].

Facial expressions can provide insight into an individual’s emotional state, and automatic facial expression analysis (AFEA) is a topic of broad research [31]. However, the contribution of pain expression is less extensive, especially for infant pain assessment. There are several researches on pain facial expression recognition in adults [19,20,23,32,33,34]. However, the methods designed for adult pain assessment may not show similar performance and may completely fail in infants for three main reasons: First, facial morphology and dynamics vary between infants and adults, as reported in [35]. Moreover, infant facial expressions include additional important units that are not present in the Facial Action Coding System. As such, NFACS (Neonatal Facial Coding System) is introduced as an extension of FACS [35,36]. Second, infants with different individual variables (such as gestational age) have different pain facial characteristics due to a less developed central nervous system [36]. Third, the preprocessing stage is more challenging in case of infants, since they are uncooperative subjects recorded in an unconstrained environment. In this paper, we focus on studies on infant pain assessment.

Brahnam et al. [37] utilized the holistic eigenfaces approach to recognize pain facial expressions in newborn babies, and compared the performances of distance-based classifier and Support Vector Machine (SVM) for pain detection. This work was extended by employing Sequential Floating Forward Selection for feature selection and Neutral Network Simultaneous Optimization Algorithm (NNSOA) for classification; an average classification rate of 90.2% was obtained [38]. Gholami et al. [39] presented Relevance Vector Machine (RVM) to assess infant pain and its intensity. The classification accuracy of RVM (85%) was found to be close to assessments by experts. Nanni et al. [40] used several histogram-based descriptors to detect infant pain facial expression, including Local Binary Pattern (LBP), Local Ternary Pattern (LTP), Elongated Ternary Pattern (ELTP), and Elongated Binary Pattern (ELBP). The highest accuracy was achieved by ELTP with Area under the Curve of Receiver Operating Characteristic Curve (AUC) score of 0.93. The above researches were conducted using the COPE database, which is the only open access infant pain database, consisting of 204 static 2D images of 26 infants photographed under pain stimuli. Static images reveal facial expressions in certain photographs, while ignoring temporal information of pain.

A few studies have recently focused on dynamic pain facial expression analysis. Fotiadou et al. [41] applied the Active Appearance Model (AAM) to extract facial features and global motion for each video frame. SVM classifier was utilized for pain detection in 15 videos of eight infants, and the AUC achieved 0.98. Zamzmi et al. [42,43] extracted pain-relevant facial features from video sequences by estimating the optical strain magnitudes corresponding to pain facial expressions. SVM and KNN (K-nearest neighbor) classifiers were employed for pain detection, and an overall accuracy of 96% was obtained.

Most AFEA systems focus on facial expression analysis in near-frontal-view of facial recordings, and very few studies investigate the effect of the profile view of a facial image [44,45]. According to clinical observations, head movements occur commonly during pain experiences. Head shaking results in multi-view faces and may lead to failure of face detection and pain recognition. Facial expression recognition for a profile-view is challenging, as a lot of the facial representation information is lost. There is no research available that investigates pain facial expression recognition performances on profile view.

According to clinical research, infants with low gestational age have less-developed central nervous systems, and show limited ability to behaviorally communicate pain in comparison to full-term or post-term infants [46]. Derbyshire [47] revealed high pain sensitivity in female adults than in males with different situational cases, while pain responses for early age infants were not found to be extremely affected by sex differences. Due to the complexity of clinical contexture, infant pain facial expression analysis is more challenging, and contextual and individual factors are worth considering (i.e., age, gender, and race).

There is growing evidence in psychological research of the fact that temporal dynamics of facial behavior (e.g., the duration of facial activity) is a critical factor in the interpretation of observed behavior [45]. In this paper, we propose a dynamic pain facial expression representation and fusion scheme for automatic infant pain assessment, by combining temporal appearance facial features and temporal geometric facial features. Different automatic pain assessment models are constructed to gain a better understanding of the various factors that influence pain reactivity in infants, including gestational age, gender, and race. Moreover, a pain assessment model based on facial profile view is also investigated. The effectiveness of a specific model constructed according to individual variables is analyzed and compared with a general model. To the best of our knowledge, this is the first study investigating infant pain recognition depending on multiple facial views and various individual variables.

## 3. Methodology

Temporal information for facial representation is crucial for emotional analysis, especially for subtle facial expressions. Static images are photographed at discrete points in time, and they only include limited facial activity information where facial changes over time are missing; for example, eye blinking, which is important for pain recognition, cannot be distinguished by static features. In this section, we describe our dynamic pain facial activity representation method, including frame-level facial configuration/texture parameters representation, and sequence-based temporal descriptors extraction. Hybrid pain facial activity representation, including facial geometric features and texture features, is studied for pain facial expression recognition. The temporal pain-related facial activity representation is the same as in our preliminary work [48]. The dimensionality of hybrid facial features is reduced by manifold learning-based Supervised Locality Preserving Projections (SLPP) method, and SVM classifier is utilized for pain recognition. Finally, the classifier outputs of multiple facial activity features are combined by decision fusion.

### 3.1. Frame-Level Hybrid Facial Representation

The facial configuration parameters are designed by a set of geometric distance parameters, which intuitively depict facial deformation during pain experiences. On the other hand, facial appearance descriptors are derived from the gradient magnitude of a set of Region around Points (RAPs) centered on key facial fiducial points. Several facial activity representation parameters are extracted from each frame image and the details are described as follows.

**Figure 1:**
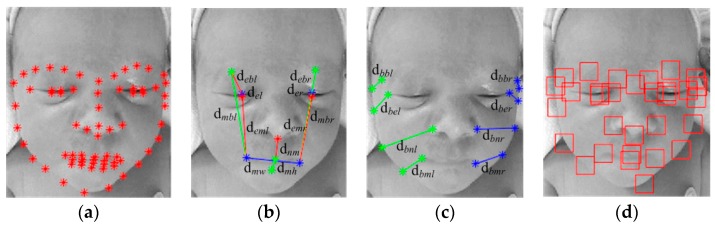
Frame-level parameters of infant pain facial expression. (**a**) Facial landmarks; (**b**) facial configuration parameters; (**c**) head pose parameters; (**d**) landmark patches.

- **Facial landmark detection:** Both facial configuration parameters and facial texture parameters are calculated on the basis of facial landmarks. We apply the popular Active Appearance Model (AAM) to detect infants’ facial landmarks (68 points) in image sequences to outline the main features of the eyebrows, eyes, nose, mouth, and face boundary (see Figure 1a). AAM combines a shape variation model with an appearance variation model in a shape-normalized frame, and is improved upon to be rapid, accurate, and robust [49].
- **Facial deformation measurement:** Several pain-related geometric distance parameters are derived for each image frame from the facial fiducial points of the eyebrows, eyes, nose, and mouth, to capture deformations of facial components. Specific facial actions have been identified in Neonatal Facial Coding System (NFCS), which is a subsystem of Facial Action Coding System (FACS), and specially designed for pain assessment for newborns to 2 months of age. The validity and reliability of the sign judgment method has been evaluated in previous studies [3]. The facial actions for infant pain assessment include brow bulge, nasolabial furrow, eye squeeze, chin quiver, open lips, lip purse, horizontal mouth stretch, vertical mouth stretch, taut tongue, and tongue protrusion [36]. Thus, distance parameters are defined by the NFCS as the Euclidean distance between key facial fiducial points, including distance between the eyebrow and the eye (*d_ebl_* and *d_ebr_*), distance between the upper eyelid and the lower eyelid (*d_el_* and *d_er_*), distance between the eyebrow and the mouth (*d_mbl_* and *d_mbr_*), distance between the eye and the mouth (*d_eml_* and *d_emr_*), distance between the nose and the mouth (*d_nm_*), and the width (*d_mw_*) and height (*d_mh_*) of the mouth. Facial deformation parameters are shown in Figure 1b.
- **Head pose movement measurement:** According to clinical observation, infants in pain usually shake their head left and right. Head movement is a useful predictor for infant pain assessment. Since there is no obvious relationship between pain occurrence/intensity and the head orientation angle, we conducted distance-based head pose parameters in a simple manner. The distance parameters include the distances between key facial components landmarks (eyebrows, eyes, nose, and mouth) and face boundary landmarks for both left and right sides of the face (*d_bbl_*, *d_bel_*, *d_bnl_*, *d_bml_, d_bbr_, d_ber_, d_bnr_, d_bmr_*). The head pose parameters are illustrated in Figure 1c.
- **Facial texture measurement:** Appearance-based facial expression features are extracted to reflect the magnitude and direction of skin surface displacement. Based on primary facial landmarks, patches with size of 32 × 32 are defined around the landmarks. A domain knowledge-based texture description method is employed by calculating the mean magnitude for each local patch. These patches cover most of the facial regions, as illustrated in Figure 1d, where obvious wrinkles usually appear between the eyebrows (eyes), the corner of eyes, nasal root, and corner of the mouth. It is a simple and low-dimensional facial texture representation comparing to generic facial features, as only one numeric parameter is obtained for each RAP, and there are 31 texture parameters for each frame image.

### 3.2. Sequence-Based Temporal Descriptors Extraction

The facial activity parameters are derived from each frame of video sequences, and they compose of several feature signals along the temporal dimension, including facial configuration (face deformation and head movement) and facial texture. A set of descriptors are extracted from these signals to represent the dynamic features for each video sequence [24].

First, the descriptor signals are temporally smoothed by Butterworth filter (first order with cutoff 1 Hz for temporal signals). Then the first and second derivation of the smoothed signals are estimated for feature description. The original descriptor signal is denoted by *x*, the smoothed signal is *s*, and its first and second derivation are denoted by *v* and *a*, respectively. A set of features are extracted from each temporal signal (i.e., *s*, *v* and *a*) to depict the characteristics of the signal variance over time for different aspects of the signal. These parameters include: (1) state parameters: maximum value, minimum value, mean value, and median value; (2) variability parameters: range, standard deviation, inter-quartile range, inter-decile range, and median absolute deviation; (3) peak parameters: instant of time when the amplitude is at its maximum; (4) duration parameters: duration of when the amplitude is greater than mean, and duration of when the amplitude is greater than the average of mean and minimum value; (5) segment parameters: number of segments where the amplitude is greater than mean value, and number of segments where the amplitude is greater than the average of mean and minimum value; (6) area parameters: area between signal and its minimum (AREA), and quotient of AREA (the difference between maximum and minimum value). The first and second derivation of the signals could be treated as the speed and acceleration of the descriptor signals, and these temporal features could describe varying information of facial activity signals, such as amplitude, speed, variability and the time interval of the facial motion. In summary, there are 16 × 3 temporal feature parameters for each facial activity signal.

According to our previous research, the dynamics of temporal facial appearance descriptors are crucial for a description of infant pain expression. LBP-TOP [50] is an effective spatio-temporal histogram representation that captures facial replacement along time. The histogram features are extracted from three orthogonal planes: X-Y (horizontal-vertical spatio), X-T (horizontal spatio-temporal), and Y-T (vertical spatio-temporal). The LBP-TOP is computed on the RAP defined by the 31 facial landmarks, as described in Section 3.1. The dimensions of the dynamic appearance feature for each video is 5487 (177 × 31), which concatenate local appearance descriptors over patches. LBP-TOP based histogram is gray invariant and rotation variant, and the dynamic facial features can tolerate complex environmental conditions to a certain degree.

### 3.3. Dimensionality Reduction Using SLPP

Locality Preserving Projections (LPP) are linear projective maps that approximate the eigenfunctions of the Laplace-Beltrami operator of the manifold. LPP aims at embedding high dimensional features into an intrinsic low-dimensional subspace, which preserves local structure by constructing an adjacency graph denoting the weight matrix. The LPP inherits the advantages of nonlinear manifold learning and provides transformation function explicitly. The weight matrix is constructed according to the distances between pairs of samples, and the criterion can be applied in an unsupervised or supervised manner. In a supervised manner, the prior class information of the samples is utilized, and the maps will preserve the class structure of the samples. More details of the supervised LPP (SLPP) can be found in [51].

The compact features extracted by SLPP are fed to a classifier to enhance classification performances, and low-dimensional features are mentioned as facial activity features in subsequent sections.

### 3.4. Classification and Decision Fusion

In our paper, we employ Support Vector Machine (SVM) for infant pain recognition. SVM is a well-applied classifier for binary classification and multi-class classification. SVM allows domain-specific selection of the kernel function and can also generalize well with few training data. Each type of facial activity feature trains a SVM classifier individually, and the output is the identification of pain state. Multiple facial activity features are then combined by the decision fusion scheme, i.e., majority voting.

Majority voting is a simple and effective decision fusion method, and fusion criterion is “more than half”. Temporal geometric and texture features are fed to the SVM classifier individually, and each output contributes one vote to the final decision; the major class in the combination is the final label. If there is a tie for different indicators, the class with the highest confidence score is chosen as the final decision of infant pain assessment.

## 4. Dataset

One of the crucial issues in evaluating the performance of a proposed multiple temporal facial activity feature fusion method is the acquisition of infant pain data. To our knowledge, the COPE database [52] is the sole public infant pain database, consisting of 204 static images of 26 infants, face photographed under pain stimuli. However, there is no video data available for research communities. In our experiments, evaluation is conducted on our Infants Pain Assessment Database (IPAD). The detailed information of the IPAD is as follows:

### 4.1. Subjects

**Figure 2:**
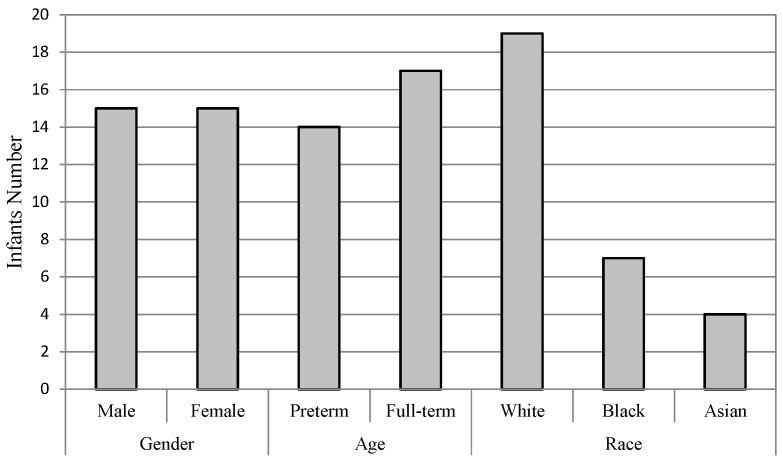
Number of infants in different groups.

A total of 31 infants were recorded during routine painful procedures such as heel lancing, which last for about 5 s, when they were hospitalized in the NICU at Tampa General Hospital. Consent was obtained from the parents of the enrolled infants. Half of the infants were male (50%). The average gestational age of the infants was 36.4 weeks, ranging from 30.4 to 40.6 (*SD* = 2.7). Infants born before 37 gestational weeks are called preterm, and full-term gestation is 37 weeks to 42 weeks. Infants were also racially diverse, with White, Black, and Asian. The distribution of infants’ various attributes is shown in Figure 2.

### 4.2. Data Acquisition and Preprocessing

The video recordings for infants were acquired by GoPro Hero3+ camera, capturing their facial expression, body movement, and sound. The camera was set in the normal clinical environment, and the videotapes recorded the infants’ spontaneous responses during acute painful treatments.

In the preprocessing step, each procedure video is segmented into seven time periods for subsequent analysis. These epochs include five minutes prior to the procedure to provide a baseline state (*T*_0_); the pain procedure period (*T*_1_), and every minute after the completion of the painful procedure for five minutes (*T*_2_~*T*_6_). Each epoch is assigned pain or no-pain, according to the indicator-based pain scale for infants.

### 4.3. Ground Truth Assessment

The Neonatal Infant Pain Scale (NIPS) [53] is a reliable and valid indicator-based pain scale for both preterm and full-term infants. Both behavioral and physiological indicators are involved, such as facial expression, crying, breathing patterns, arm movements, leg movements, and state of arousal. Each indicator is scored with 0 or 1, except “cry” which has three response categories, i.e., 0, 1, and 2. Total pain score, ranging from 0 to 7, is obtained by summing all the indicator scores. The infants’ pain levels are divided into three groups, which are determined by the total pain scores, that is, no pain (0~2), moderate pain (3~4), or severe pain (>4).

The nurses rate the severity of the infants’ pain indicators at one-minute intervals during the pain procedure, and the total pain scores are treated as the ground truth for infant pain assessment. The label of pain (3~7)/no-pain (0~2) for each sample is utilized to evaluate the performance of our algorithms.

## 5. Experimental Results and Discussion

The general performance of our automatic infant pain assessment scheme is evaluated in this section. The temporal facial geometric features include facial configuration representation (DG*_DisFace_*) and head pose representation (DG*_DisPose_*). The temporal facial texture feature of gradient representation is denoted by DA*_Gradient_*, and LBP-TOP representation is denoted by DA*_LBP-TOP_*. The facial features are low-dimensional data extracted by SLPP and employed to train SVM classifiers individually. The output of the classifier is a binary label of pain or no-pain.

The data set is split into two subsets: one subset is a training set used to develop a classifier model, and the other is a testing set used to evaluate the generalization performance of the classifier model. Due to the limitation of infants’ pain samples, it is fair to conduct cross-validation as a powerful general technique. In our experiments, the Leave-One-Subject-Out cross validation is conducted for subject-independent evaluation.

### 5.1. Comparison of Multi-Feature Fusion

The dynamic geometric features and dynamic appearance features are combined through a decision fusion scheme, and the overall accuracies are compared in Table 1. The differences between recognition accuracies achieved by multiple facial features are identified by a statistical significance test known as *t*-test (Table 2).

Generally, the single dynamic appearance features outperform the single dynamic geometric features, especially when compared to DG*_DisPose_*. The accuracy of dynamic appearance feature DA*_LBP-TOP_* is higher than that of DA*_Gradient_*, and the T-test indicates that there are no significant differences between these two types of facial features. On the other hand, the computational complexity of DA*_Gradient_* is lower than that of DA*_LBP-TOP_*, depending on the original feature dimensions (DA*_LBP-TOP_*: 5487 = 177 × 31, DA*_Gradient_*: 1488 = 16 × 3 × 31, for each video instance).

Although the performance of a single head pose-related feature is not satisfied, it enhances the accuracy of multi-feature fusion to a certain degree. Difference analysis shows that there are significant differences between single (DG*_DisPose_*) and multi-features, including two and three feature combinations (*p* < 0.05). It can be seen that the accuracy of a two-feature combination (geometry and appearance) is significantly higher than that of corresponding single features. The three-feature combinations of (DG*_Dis__Face_* & DG*_DisPose_* & DA*_Gradient_*) and (DG*_Dis__Face_* & DG*_DisPose_* & DA*_LBP-TOP_*) achieve the highest recognition accuracy of 88.48% and 89.33%, respectively. In spite of no significant difference between three-feature combinations and two-feature combinations in the statistical *t*-test, the recognition accuracy of (DG*_Dis__Face_* & DG*_DisPose_* & DA*_LBP-TOP_*) is approximately more than 2% higher than that of (DG*_Dis__Pose_* & DA*_LBP-TOP_*). Multiple dynamic facial features provide sufficient information for infant pain assessment and are tolerant to missing data caused by hospitalization environments, such as occlusions. More details of the comparison of multiple dynamic facial features, including feature fusion and decision fusion, are stated in our previous study [48]. Based on these observations, we apply the three-feature decision fusion in subsequent sections for further analysis.

### 5.2. Pain Assessment in Profile View

Machine analysis of facial behavior has achieved good recognition accuracies, but they perform only on frontal faces or near-frontal faces, where the individuals face the camera and do not change their head pose three-dimensionally. However, according to clinical observation, head shaking occurs frequently during painful procedures, leading to out-of-plane rotation, which is challenging for pain detection.

**Figure 3:**
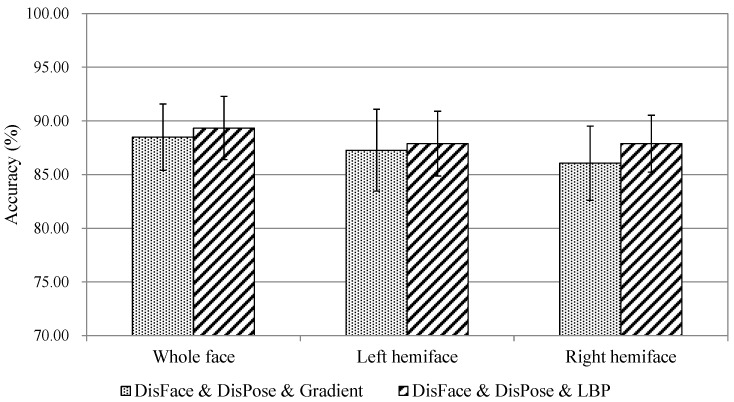
Overall accuracy comparison of whole face and hemiface model.

In this section, we investigate infant pain assessment by carrying out hemiface facial feature-based fusion and classification. The temporal dynamics of geometric and appearance features of an infant’s left and right side of the face are employed for pain assessment; the results are demonstrated in Figure 3 with confidence intervals denoted by line. The best recognition accuracy is 87.88% of the left hemiface, and is equal to the best recognition rate of the right hemiface, both of which are achieved by (DG*_Dis__Face_* & DG*_DisPose_* & DA*_LBP-TOP_*). This is consistent with the evidence that spontaneous facial expressions are more symmetrical (involving left hemiface and right hemiface) than deliberate expressions on request [54]. The accuracies of profile evaluation drop by around 1.4%, compared to whole face evaluation. We think it is acceptable, as half of the facial behavior information is missing when hemiface-based pain assessment is applied. The statistical *t*-test indicates that there is no significant difference (*p* < 0.05) between whole face accuracy and hemiface accuracy. It shows that facial symmetry has sufficient discriminating power for infant pain assessment.

**Figure 4:**
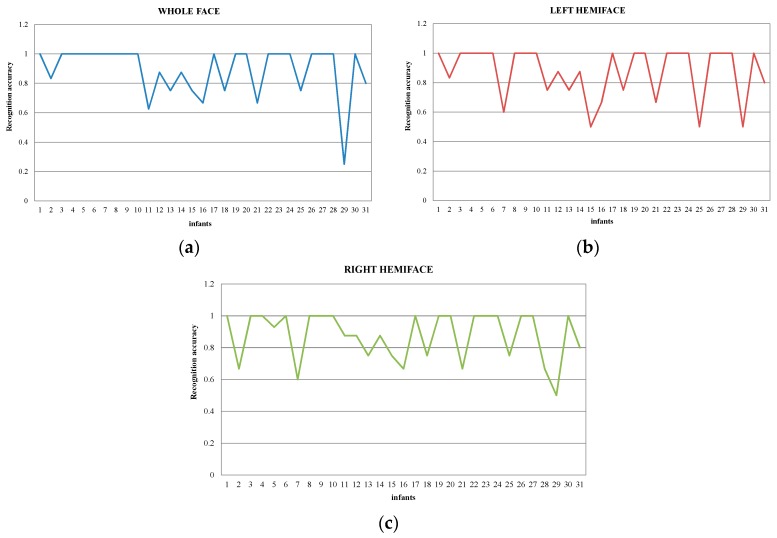
Individual accuracies of whole face and hemiface models; (**a**) Whole face (**b**) Left hemiface (**c**) Right hemiface.

Furthermore, the performances of individuals are investigated and the accuracies of (DG*_Dis__Face_* & DG*_DisPose_* & DA*_LBP-TOP_*) are shown in Figure 4. A majority of the infants achieve accuracy of 100%, while only four infants get low accuracies (<70%) in whole face-based pain recognition. The results of hemiface-based pain recognition are similar, with bad performances for some of the infants. For infants 11, 16, and 21, the no-pain instances are misclassified as pain, due to poor light conditions (dark) and frequent head shaking during a no-pain procedure. The pain instances of infants 29 are misclassified as non-pain, as the infants do not show pain expression during the procedure. As all the infants’ videos are captured in a real NICU, the proposed dynamic facial representation and fusion scheme was able to deal with complex conditions, such as low quality and occlusion.

### 5.3. Individual Variability Analysis

Studies demonstrated that infants’ biobehavioral pain responses vary due to limited neurological development. For a better understanding of the factors that influence pain reactivity in infants, it is necessary to analyze the effects of individual variables, such as gestational age, gender, and race. In this section, the proposed infant pain assessment method is evaluated for these factors. The total IPAD database is divided into diverse groups according to individual variables, and the pain recognition model is constructed for factor-related subsets, respectively (specific model). The experimental results of the specific model are compared to corresponding results of the general model, which is constructed by all the 31 infants. Both overall accuracies and AUC (area under the Receiver Operating Characteristic (ROC) curve) scores are demonstrated in Table 3 for three-feature fusion of (DG*_Dis__Face_* & DG*_DisPose_* & DA*_Gradient_*) and (DG*_Dis__Face_* & DG*_DisPose_* & DA*_LBP-TOP_*). The best results of overall accuracies are marked in gray, and the best results of AUC scores are marked in green. The experiments are organized into three blocks according to main individual variables: gender, gestational age, and race.

**Figure 5:**
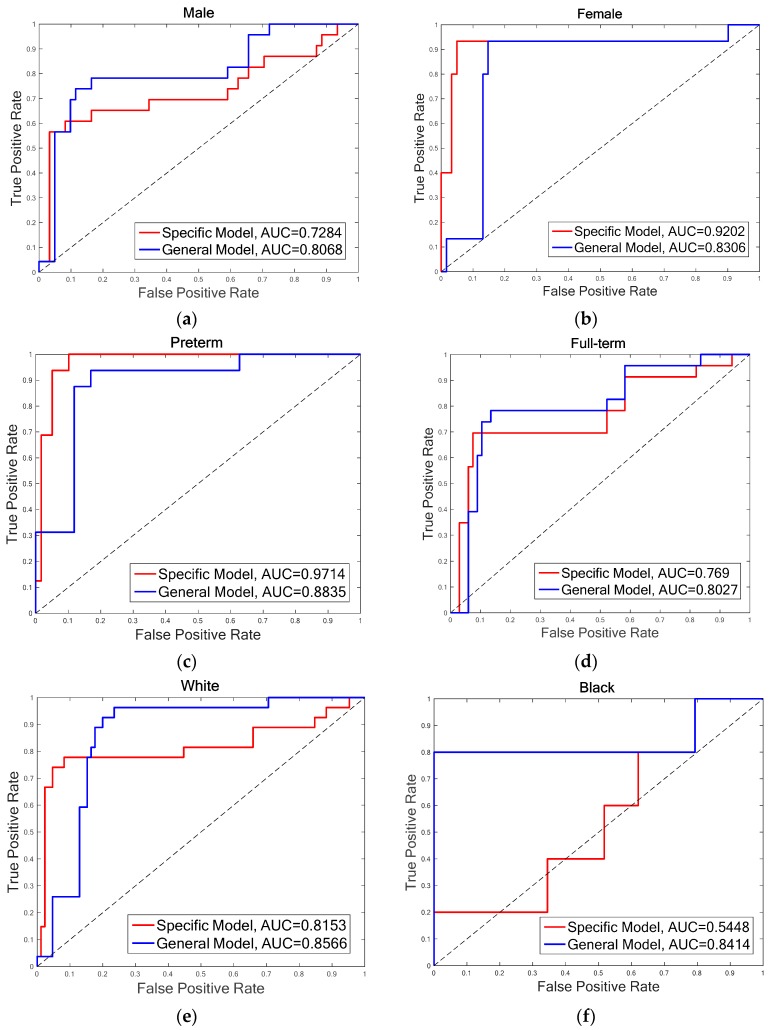
ROC curves for different individual groups with FDL (**a**) Male (**b**) Female (**c**) Preterm (**d**) Full-term (**e**) White (**f**) Black.

- **Gender:** The infants are divided into two groups of male and female. There are 15 infants in each group, as one infant’s gender information is not recorded on the score sheet. For the male group, the overall accuracies of both FDG (DG*_DisFace_* & DG*_DisPose_* & DA*_Gradient_*) (88.39%) and FDL (DG*_DisFace_* & DG*_DisPose_* & DA*_LBP-TOP_*) (85.86%) achieved by the specific model are higher than that of the general model (FDG: 82.14%, FDL: 84.52%), while the overall accuracies of female group are just the opposite, i.e., higher accuracies of 94.74% (FDG and FDL) are obtained by the general model. However, no significant differences are found between the specific model and the general model for overall accuracies. Moreover, due to the bias of data distribution, it is also useful to analyze the AUC results. According to the AUC scores, the general model performs better than the specific model. Table 3 shows that although the accuracies of the specific model for the male group are higher, the AUC scores of the general model are much better for both male and female groups, except for the FDL feature in the female group. The ROCs of FDL for the specific model and the general model are illustrated in Figure 5a,b.
- **Gestational age:** The database is divided into two groups of infants with different gestational age, that is, preterm (<37 weeks) and full-term (37 weeks to 42 weeks). For the preterm group, the highest accuracy is 96%, which is achieved by FDG in the specific model and FDL in the general model, while the AUC scores obtained in the specific model are higher than that of the general model for both FDG (0.9894) and FDL (0.9714). The statistical analysis indicates that there is no difference in the other groups in terms of the relationship with the general model. For the full-term group, the highest accuracy (84.44%) and AUC scores (0.8047) are obtained by general model. The general model outperforms the specific model in the aspect of both overall accuracy and AUC score. The ROCs of FDL for two models of the preterm and full-term are shown respectively in Figure 5c,d.
- **Race:** The infants belonged to three races: White, Black, and Asian. Only four infants were recorded as Asian; thus, it is not possible to construct an Asian pain assessment model. We examine the effectiveness of specific models for the White group and the Black group, individually. Table 3 demonstrates that the best overall accuracy of the White group is 89.29% (for both FDG and FDL in the general model), and the highest AUC score of 0.9111 is obtained in the general model by FDG. Similar results were found for the Black group; the highest accuracy is 97.06% and AUC score is 0.8414, which was achieved by FDL in the general model. The statistical analysis does not find significant differences between the overall accuracies of the specific model and the general model. More details are demonstrated in Figure 5e,f through the ROCs of diverse models for two infant groups, depending on race.

In conclusion, the general model performs better than the specific model for various infant groups of male, female, full-term, white and black. Higher accuracies are obtained in the general model, except for the male group, although no significant difference is found between the two types of models. The best-performed AUC scores were achieved in the general model for the individual variable groups mentioned above. Since the binary labeled infant data is biased, it is valuable to take into consideration the AUC and ROC results. However, for the “preterm” group, better performance is obtained by the specific model for both accuracy and AUC. According to clinical research, infants with low gestational age were not able to express their feelings in the same way as fully developed infants do, and they had fewer behavioral responses. This may be the reason why the specific model is better for preterm infants’ pain detection. Other infant groups do not show such requirements of constructing a pain assessment model by homogeneous data. Therefore, gestational age is the most influencing factor for pain assessment, and it is necessary to construct a specific model depending on gestational age. Besides, it is worth mentioning that the sample size of IPAD (general model) is still small, leading to an even-smaller sample size in specific models. Therefore, it may lead to erroneous conclusions when comparing the specific model to the general model. However, this study provides a pioneer view of the influence factor investigation for automatic infant pain assessment, and we would like to continue collecting data and evaluating our method on a larger dataset.

## 6. Conclusions

In this study, we propose the dynamics of temporal facial representations of both geometry and appearance; decision fusion is utilized for multi-feature combination. Facial configuration descriptors, head pose descriptors, and gradient descriptors are extracted from the time series of frame-level features; and temporal texture descriptor LBP-TOP is employed to describe facial changes over time. The dynamic facial representation and fusion scheme is successfully applied for infant pain assessment. We also investigate the effects of various factors that influence pain reactivity in infants, such as individual variables of gestational age, gender, and race. Different automatic infant pain assessment models are constructed depending on various influence factors, as well as profile views of faces which affect the model ability of pain recognition. It can be concluded that the profile-based infant pain assessment is feasible, as its performance is almost as good as the performance of the whole face. The proposed automatic pain assessment scheme is employed on different infant groups, depending on individual variables; and the comparison between the specific model and the general model shows no significant difference between the overall accuracies of these two model types. Moreover, the results of AUC scores are also compared and the general model outperforms the specific model for male, female, full-term, white, and black infant groups. However, for the “preterm” group, better performance is obtained by the specific model for both accuracy and AUC score. Therefore, gestational age is the most influencing factor for infant pain assessment, and it is necessary to construct specific models depending on gestational age. This is mainly because of the limited ability of behavioral communication for infants with low gestational age.

Due to limited sample size of IPAD, some of the conclusions may not be general, when comparing the specific model to the general model, as the former has an even-smaller sample size. On the other hand, there is no control procedure for pain data acquisition. Spontaneous responses are recorded in a normal clinical environment. The labels of the instants are assigned according to a behavioral indicator scale, and it is treated as a gold-standard for infant pain assessment. In terms of future research possibilities, we would like to collect infants’ pain data continuously by taking into consideration a control procedure and evaluate our method on a larger dataset.

## Figures and Tables

**Table 1:** Comparison of diverse dynamic facial feature combination (%).

	DG*_DisFace_*	DG*_DisPose_*	DA*_Gradient_*	DA*_LBP-TOP_*	Decision Fusion
Single feature	√				87.98
		√			79.97
			√		85.38
				√	87.66
Two-feature	√	√			87.67
	√		√		88.36
		√	√		82.55
	√			√	87.88
		√		√	85.51
Three-feature	√	√	√		88.48
	√	√		√	89.33

**Table 2:** Difference analysis of single facial feature and multiple facial features.

	DG_*DisFace*_	DG_*DisPose*_	DA_*Gradient*_	DA_*LBP-TOP*_	DG_*DisFace*_ & DG_*DisPose*_	DG_*DisFace*_ & DA_*Gradient*_	DG_*DisPose*_ & DA_*Gradient*_	DG_*DisFace*_ & DA_*LBP-TOP*_	DG_*DisPose*_ & DA_*LBP-TOP*_	DG_*DisFace*_ & DG_*DisPose*_ & DA_*Gradient*_
**DG*_DisPose_***	**2.922 (0.004)**									
**DA*_Gradient_***	1.345 (0.181)	1.518 (0.131)								
**DA*_LBP-TOP_***	1.028 (0.306)	1.227 (0.222)	0.377 (0.707)							
**DG*_DisFace_* & DG*_DisPose_***	0.000 (0.999)	**2.922 (0.004)**	1.345 (0.181)	**2.573 (0.011)**						
**DG*_DisFace_* & DA*_Gradient_***	0.446 (0.656)	**2.647 (0.009)**	1.294 (0.198)	1.958 (0.052)	0.446 (0.656)					
**DG*_DisPose_* & DA*_Gradient_***	1.000 (0.319)	**2.226 (0.027)**	0.904 (0.367)	1.214 (0.226)	1.000 (0.319)	1.000 (0.319)				
**DG*_DisFace_* & DA*_LBP-TOP_***	1.419 (0.158)	**2.603 (0.010)**	0.894 (0.373)	**2.144 (0.033)**	1.419 (0.158)	0.446 (0.656)	0.332 (0.740)			
**DG*_DisPose_* & DA*_LBP-TOP_***	1.345 (0.181)	**2.324 (0.021)**	0.624 (0.533)	**2.264 (0.025)**	1.345 (0.181)	0.706 (0.481)	0.000 (0.999)	1.419 (0.158)		
**DG*_DisFace_* & DG*_DisPose_* & DA*_Gradient_***	0.000 (0.999)	**2.922 (0.004)**	1.345 (0.181)	**2.573 (0.011)**	0.000 (0.999)	0.446 (0.656)	1.000 (0.319)	1.345 (0.181)	0.576 (0.565)	
**DG*_DisFace_* & DG*_DisPose_* & DA*_LBP-TOP_***	0.928 (0.355)	**2.769 (0.006)**	1.728 (0.086)	**2.367 (0.019)**	0.928 (0.355)	1.096 (0.275)	1.419 (0.158)	0.928 (0.355)	1.351 (0.179)	1.518 (0.131)

Note: The values outside the parentheses are *t*-values, and the values in the parentheses are *p*-values. Significant differences are highlighted in bold (*p* < 0.05).

**Table 3:** Pain assessment results of various factor groups.

			Gender		Age		Race	
			Male	Female	Preterm	Term	White	Black
FDG	Specific	Rate	88.39	89.17	96.00	81.11	89.29	94.12
		AUC	0.7833	0.7738	0.9894	0.7242	0.8436	0.7379
	General	Rate	82.14	94.74	93.33	84.44	89.29	91.18
		AUC	0.8054	0.9224	0.9788	0.8047	0.9111	0.7862
FDL	Specific	Rate	85.86	91.48	93.33	83.33	89.29	88.24
		AUC	0.7284	0.9202	0.9714	0.7690	0.8153	0.5448
	General	Rate	84.52	94.74	96.00	84.44	89.29	97.06
		AUC	0.8068	0.8306	0.8835	0.8027	0.8566	0.8414

Note: FDG means “DG*_DisFace_* & DG*_DisPose_* & DA*_Gradient_*”; FDL means “DG*_DisFace_* & DG*_DisPose_* & DA*_LBP-TOP_*”. AUC means area under Receiver Operating Characteristic (ROC) Curve.
